# Effects and Impact of Selenium on Human Health, A Review

**DOI:** 10.3390/molecules30010050

**Published:** 2024-12-26

**Authors:** Song Bai, Miaohe Zhang, Shouying Tang, Miao Li, Rong Wu, Suran Wan, Lijun Chen, Xian Wei, Shuang Feng

**Affiliations:** 1 Guizhou Industry Polytechnic College, Guiyang 550008, China; basonmail@163.com (S.B.); lmiaoooo@163.com (M.L.); wurong199706@163.com (R.W.); wansuran1213@163.com (S.W.); clj102128@163.com (L.C.); 2 School of Chemical Engineering, Guizhou Institute of Technology, Guiyang 550003, China; zmh_870927@163.com (M.Z.); weixian424@sohu.com (X.W.); feng_shuang1989@sina.com (S.F.); 3 National Key Laboratory of Green Pesticide, Key Laboratory of Green Pesticide and Agricultural Bioengineering, Ministry of Education, Guizhou University, Guiyang 550025, China

**Keywords:** selenium, selenoproteomics, glutathione peroxidases, thioredoxin reductases, iodothyronine deiodinases, human health

## Abstract

Selenium (Se) is an essential trace element that is crucial for human health. As a key component of various enzymes and proteins, selenium primarily exerts its biological functions in the form of selenoproteins within the body. Currently, over 30 types of selenoproteins have been identified, with more than 20 of them containing selenocysteine residues. Among these, glutathione peroxidases (GPXs), thioredoxin reductases (TrxRs), and iodothyronine deiodinases (DIOs) have been widely studied. Selenium boasts numerous biological functions, including antioxidant properties, immune system enhancement, thyroid function regulation, anti-cancer effects, cardiovascular protection, reproductive capability improvement, and anti-inflammatory activity. Despite its critical importance to human health, the range between selenium’s nutritional and toxic doses is very narrow. Insufficient daily selenium intake can lead to selenium deficiency, while excessive intake carries the risk of selenium toxicity. Therefore, selenium intake must be controlled within a relatively precise range. This article reviews the distribution and intake of selenium, as well as its absorption and metabolism mechanisms in the human body. It also explores the multiple biological functions and mechanisms of selenium in maintaining human health. The aim is to provide new insights and evidence for further elucidating the role of selenium and selenoproteins in health maintenance, as well as for future nutritional guidelines and public health policies.

## 1. Introduction

Selenium (Se) is a trace element that is essential for both humans and animals. It is widely distributed in various tissues and organs of the human body and animals. Selenium is closely related to human health and is involved in regulating a variety of physiological functions. Selenium was first discovered by the Swedish chemist Berzelius in 1817 in the tailings of sulfuric acid production and was long considered a toxic element. It was not until 1957 that Schwarz and Foltz first demonstrated that selenium is an essential nutrient for animals [1]. In 1973, Rotruck et al. [2] discovered and confirmed that selenium is a component of glutathione peroxidase in humans and animals. In 1979, the Chinese Keshan Disease Research Group proved that selenium deficiency is a necessary condition for Keshan disease. Selenium is not only a component of several important enzymes, such as glutathione peroxidases (GPXs), thioredoxin reductases (TrxRs), and iodothyronine deiodinases (DIOs), but it also has multiple biological functions, including antioxidant properties, regulation of thyroid function, anti-cancer effects, enhanced immunity, protection of the cardiovascular system, enhancement of reproductive capacity, and anti-inflammatory effects [3,4,5,6,7,8,9,10,11]. Despite its critical importance to human health, the range between nutritional and toxic doses of selenium in the human body is very narrow. Insufficient daily selenium intake can lead to selenium deficiency, while excessive daily selenium intake can lead to selenium poisoning [12]. Severe selenium deficiency can lead to the occurrence of diseases such as Keshan disease, Kaschin–Beck disease, and White Muscle Disease [13,14,15,16]. On the other hand, excessive selenium intake can lead to selenium poisoning, which includes acute selenium poisoning and chronic selenium poisoning. Acute selenium poisoning refers to acute toxicity caused by the ingestion of a large dose of selenium in a short period of time, with symptoms such as respiratory distress, ataxia, diarrhea, vomiting, abdominal pain, and even death. Chronic selenium poisoning refers to cumulative toxicity caused by long-term low-dose selenium intake, with symptoms including fatigue, depression, garlic-like breath odor, anemia, reduced food intake, hair loss, nail damage, hoof rot, growth retardation, and liver cirrhosis [17,18,19]. Furthermore, the human body does not have the ability to store selenium for long periods, necessitating continuous intake through the daily diet. This means that selenium intake must be maintained within a relatively precise range.

The daily dietary intake recommendations for selenium are not standardized globally, and different countries and organizations have varying guidelines. In 1987, the National Health and Medical Research Council of Australia recommended selenium intakes of 80 μg for adult men and 70 μg for adult women, making Australia the first country to set official selenium intake recommendations [20]. The U.S. National Research Council recommended a daily intake of 70 μg for adult men and 55 μg for adult women [21]. In 1996, the World Health Organization released a more standardized guideline, suggesting that adult men and women should have daily intakes of 40 μg and 30 μg, respectively. This recommendation was based on maintaining two-thirds of GPX activity in the body and ensuring adequate selenium reserves [22]. In China, to prevent Keshan disease, dietary surveys in 1987 recommended a minimum selenium intake of 19 μg for adult men and 14 μg for adult women [23]. By 2000, the Chinese Nutrition Society proposed a minimum daily selenium requirement of 41 μg for adults, a recommended intake of 50 μg, and set the maximum tolerable limit at 400 μg per day [24].

**Figure 1:**
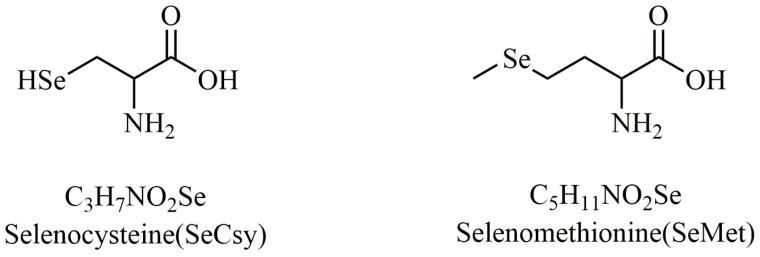
Structural formulas of selenocysteine (SeCys) and selenomethionine (SeMet).

Selenium in nature primarily exists in three forms (elemental selenium, inorganic selenium, and organic selenium). Elemental selenium is difficult for organisms to absorb and utilize. Inorganic selenium exists as selenide (Se^2+^), selenite (Se^4+^), or selenate (Se^6+^), with low bioavailability. The organic selenium present in organisms mainly includes two categories: one category consists of selenium-containing amino acids, such as selenocysteine (SeCsy) and selenomethionine (SeMet); the other category includes selenium-containing proteins, where selenium predominantly exists in the form of selenocysteine and selenomethionine residues, possessing certain physiological activities closely related to metabolism, and the structural formulas of selenocysteine and selenomethionine are in Figure 1. Currently, the known physiological functions of selenium in the human body are primarily associated with selenoproteins containing selenocysteine residues, with over 25 types identified [25]. Among these selenoproteins, glutathione peroxidase (GPX) is the most widely studied and significant. This article mainly reviews the distribution and intake of selenium, its absorption and metabolism mechanisms in the human body, and its multiple biological roles in maintaining human health. The aim is to further elucidate the role of selenium and selenoproteins in maintaining human health, providing new insights and foundations for future nutritional guidance and public health policies.

## 2. Distribution and Intake of Selenium

**Figure 2:**
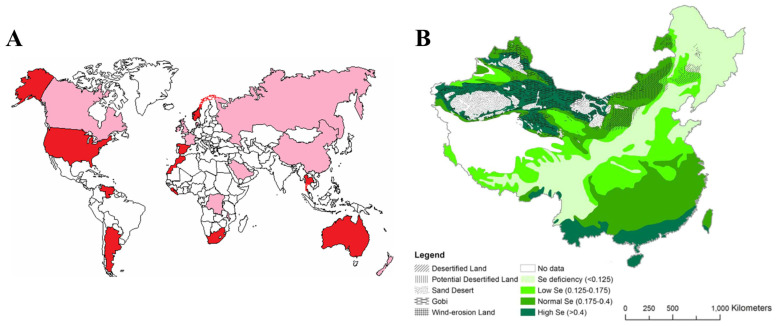
Distribution of selenium [35,36]. (**A**) Selenium distribution at the global scale: in the world map, red indicates sufficient selenium, pink indicates selenium deficiency, and white indicates no selenium content data. (**B**) Distribution pattern of soil selenium concentration in China.

The selenium in the human body primarily comes from dietary sources, and the selenium in food mainly originates from the soil. The presence and concentration of selenium in the soil largely depend on the parent material of soil formation [26]. Rock erosion is the main source of selenium deposition in the soil, and the total selenium content in rocks accounts for about 40% of the total selenium content in the Earth’s crust. Selenium is mainly found in sandstone, quartzite, and limestone [27,28]. Globally, the selenium content in most soils ranges from 0.01 to 2 mg/kg, with an average selenium content of 0.4 mg/kg [29,30]. However, soil selenium concentrations associated with specific geological features can be as high as 1200 mg/kg [31]. The distribution of selenium in the soil is extremely uneven, with significant differences in soil selenium content between different countries and even within different regions of the same country. Selenium-rich soils are widely distributed in the great plains of China, the United States, Canada, South America, Australia, India, and Russia [32]. About 80% of the world’s total selenium reserves are found in Peru, China, Chile, the United States, Canada, Zambia, the Philippines, the Democratic Republic of the Congo, Australia, and New Guinea [33]. According to data released by the World Health Organization, currently, more than 40 countries and regions globally, involving 500 million to 1 billion people, are in various states of selenium deficiency [32,34]. The distribution of selenium in the world and in China is shown in Figure 2, and the selenium content in the soil is shown in Table 1.

**Table 1:** Selenium content in soil.

Country (Region)	Sample Size	Content (mg/kg)		Remark
		Range	Mean	
Global [37]	/	/	0.4	/
USA [38,39]	910	<0.1–4.32	0.31	/
		1–10	/	Selenium-rich area
Canada [40]	173	0.03–2	0.26	/
Japan [41]	180	0.05–2.8	0.43	Agricultural soil
			0.51	
India [39]	/	0.025–0.71	/	Selenium-deficient area
	/	1–20	/	Selenium-rich area
Brazil [42]		0–2.14	/	/
Spain [43]	490	0.003–2.7	0.4	Region of Murcia
Greece [39]	/	0.05–0.10	/	Selenium-deficient area
		>0.2	/	Selenium-sufficient area
Belgium [44]	539	0.14–0.70	/	Agricultural soil
UK [45]		0.10–4	/	/
Netherlands [46]	42	0.12–1.97	0.62	Grassland
	41	0.20–1.20	0.53	Cultivated land
Scotland [47]	661	<0.06–19.2	1.04	/
Sweden [47]	5170	<0.05–13.3	0.30	/
New Zealand [39]	/	0.1–4	/	/
Scandinavian Peninsula [48]	/	0.42–0.57	/	/
Denmark [49]	/	0.14–0.52	/	/
Norway [37]	/	3–6	/	/
Pakistan [50]	/	0.041	/	/
Canada [51]	/	0.30	/	/
Iran [52]	/	0.45	/	/
Turkey [53]	/	0.9	/	/
Australia [54]	/	<0.2	/	/
China [36]	/	0.058	/	/

“/” no date available.

**Figure 3:**
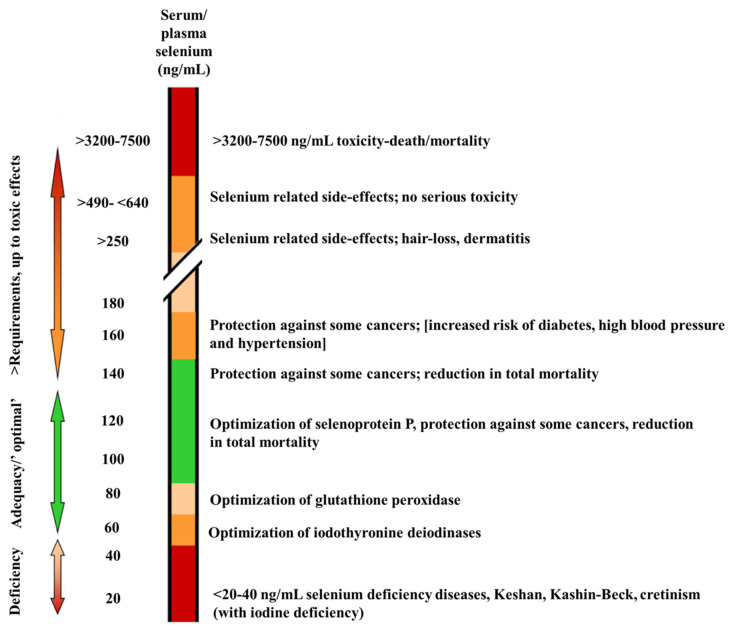
The concentration of selenium in blood and human health (range of blood selenium concentrations with possible related health effects from deficiency to toxicity) [55].

The selenium ingested from food is absorbed by the digestive system and then transported through the bloodstream to various cells throughout the body, where it participates in multiple biological processes such as antioxidation and immune enhancement. The appropriate range of selenium content in human blood is 80–140 ng/mL [55]; levels that are too high or too low can respectively lead to selenium toxicity or selenium deficiency symptoms. The range of blood selenium concentrations from deficiency to potentially health-related effects is shown in Figure 3. The primary dietary sources of selenium include seafood, such as fish and shellfish, which have higher selenium contents due to living in selenium-rich seawater [56,57]. Additionally, meats (especially liver and kidneys), edible fungi, poultry eggs, broccoli, whole grains, purple sweet potatoes, garlic, and nuts (particularly Brazil nuts) are also good sources of selenium [58].

Nutritionists advocate for supplementation with organic selenium through selenium-fortified foods, such as selenium-rich rice, selenium-rich eggs, selenium-rich mushrooms, selenium-rich tea, selenium-enriched malt, selenopolysaccharides, and selenium yeast, etc. [59,60]. The selenium content in some foods is shown in Table 2, which shows that the selenium content of Brazil nuts is relatively high, and the consumption of Brazil nuts is an efficient method of selenium supplementation. The variation in soil selenium content across different regions may lead to differences in the selenium content of the same food in different areas. Consequently, selenium intake varies between countries. The selenium intake of different countries is shown in Table 3.

The human body’s selenium requirements also vary according to different age stages. Infants, children, adults, and the elderly have distinct selenium needs due to differences in their physiological and metabolic characteristics. For example, children and adolescents need to increase their selenium intake during growth and development to support rapid physical and brain development. Adults require adequate selenium to maintain immune system function and antioxidant defenses, while the elderly may need to adjust their selenium intake due to decreased digestive and absorption capabilities. Therefore, understanding and adjusting selenium intake according to different age stages is important for maintaining health.

The estimated average requirement (EAR), recommended nutrient intake (RNI), and upper tolerable intake (UL) for selenium, as recommended by the Chinese Nutrition Society for different populations, are shown in Table 4. The daily selenium intake recommended by other countries and the World Health Organization (WHO) can be found in Table 5.

## 3. Absorption and Metabolism of Selenium in the Human Body

The absorption of selenium mainly occurs in the duodenum, with a small amount being absorbed in the small intestine and other areas [75,76,77]. Its metabolism primarily depends on the liver. Selenium binds with plasma albumin and is transported through the bloodstream to various tissues; it is then incorporated into tissue proteins in the forms of selenocysteine and selenomethionine.

Dietary selenium includes inorganic and organic selenium, and different forms of selenium have different absorption mechanisms. Overall, the human body absorbs organic selenium more efficiently compared to inorganic selenium. Dietary inorganic selenium primarily includes selenite (Se^4+^) and selenate (Se^6+^). Selenate is converted into adenosine 5′-selenophosphate through ATPase-mediated activation and then non-enzymatically reduced to selenite via glutathione (GSH) [78]. Selenite can be directly converted into hydrogen selenide (H_2_Se) under the action of thioredoxin reductase (TrxR) or sequentially converted into GSSeGS and GSSeH under the action of GSH and GPX, eventually forming H_2_Se [79,80].

**Figure 4:**
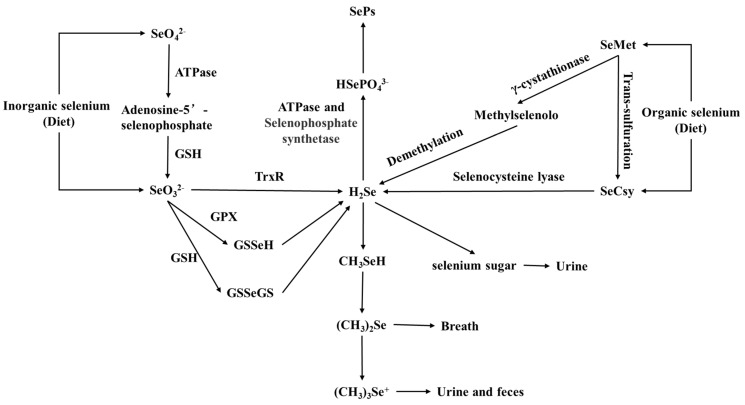
Metabolic pathway of selenium.

Dietary organic selenium mainly includes selenomethionine (SeMet) and selenocysteine (SeCys). SeMet can be non-specifically converted into methylselenol (CH_3_SeH) through cystathionine γ-lyase, which is then demethylated to produce H_2_Se [81]. Additionally, SeMet can also be converted into the intermediate product SeCys through the transsulfuration pathway. SeCys can be decomposed by selenocysteine lyase to produce H_2_Se. H_2_Se continues to be converted in two main pathways: first, under the action of ATP and selenophosphate synthetase, it is converted into selenophosphate (HSePO_4_^3−^), which is used for the generation of selenoproteins (SePs) [81]; second, it is converted into selenosugars, methylselenide (CH_3_SeH), dimethylselenide ((CH_3_)_2_Se), and trimethylselenonium ion ((CH_3_)_3_Se^+^), which are primarily excreted through urine, feces, and respiration, with a small portion being excreted through sweat [82]. When selenium intake is excessive, the liver stores selenium as glutathione peroxidase 1 (GPX1) or directly converts it into selenosugars or selenium ions for excretion. Figure 4 shows the entire metabolic process of organic and inorganic selenium from food intake to excretion.

## 4. The Biological Functions of Selenium

Selenium has many important biological functions, such as antioxidation, promoting the synthesis of thyroid hormones, exhibiting anti-cancer properties, boosting immunity, protecting the cardiovascular system, enhancing reproductive ability, and having anti-inflammatory effects [83]. However, many of selenium’s functions are manifested through different selenoproteins. Researchers have discovered more than 30 selenoproteins, but the biological functions of only a portion of these have been clearly explained. Currently, only selenoproteins containing selenocysteine residues, of which there are more than 25 (as shown in Table 6), are known to exert physiological functions in the human body.

Based on the position of selenocysteine within the polypeptide, selenoproteins can be categorized into two types: the first type, where selenocysteine is located at the N-terminus of the functional group, includes examples such as GPX, selenoprotein P, and selenoprotein W. The second type, where selenocysteine is located at the C-terminus of the peptide sequence, includes thioredoxin reductases (TrxRs), which are widely present in the organism. Among selenoproteins, the most extensively studied and important one is GPX, an essential peroxidase enzyme widely present in the body. The active center of GPX is selenocysteine, and its activity level reflects the selenium status in the body.

Currently, there are at least six types of GPXs (GPX1–GPX6). They are primarily found in the cytosol (GPX1), gastrointestinal tract and plasma (GPX2, GPX3), cell membranes (GPX4), and epididymal tissue (GPX5), while the newly discovered GPX6 is present in olfactory epithelial cells and embryonic tissue. All except GPX5 exhibit strong antioxidant activity [84,85].

**Table 6:** Human selenoproteomics.

Selenoprotein	Abbreviation	Function	Sec Location in Protein [25]	Length of Protein [25]
Glutathione peroxidase 1	GPX1	Exists in the cytoplasm, reduces cellular H_2_O_2_ [86,87].	47	201
Glutathione peroxidase 2	GPX2	Present in the gastrointestinal tract, reduces peroxide in gut [88,89].	40	190
Glutathione Peroxidase 3	GPX3	Present in plasma, reduces peroxide in blood [90,91].	73	226
Glutathione Peroxidase 4	GPX4	The enzyme, an anti-oxidative lipid repair enzyme, is localized to the cytosol, mitochondria, and nucleus. It reduces hydrogen peroxide radicals and lipid peroxides to water and lipid alcohols and prevents iron-induced cellular ferroptosis [92,93].	73	197
Glutathione Peroxidase 5	GPX5	Present in epididymal tissue [25].	Unknown	Unknown
Glutathione Peroxidase 6	GPX6	Present in olfactory epithelial cells and placental tissue [94].	73	221
Thioredoxin reductase 1	TXNRD1, TrxR1, TR1	Localized to cytoplasm and nucleus and regenerates reduced thioredoxin [95].	498	499
Thioredoxin reductase 2	TXNRD2, TrxR2, TR3	Localized to mitochondria and regenerates reduced thioredoxin [96].	655	656
Thioredoxin reductase 3	TXNRD3, TrxR3, TR2, TGR	Testes-specific expression, which regenerates reduced thioredoxin [97].	522	523
Methionine-R-sulfoxide reductase B1	MSRB1, SELR, SELX	Regulator of Factin repolymerization in macrophages during innate immune response, which works in concert with MICALs to reduce oxidated methionine (R)-sulfoxide (Met-RO) back to methionine [98,99].	95	116
Selenophosphate synthetase 2	SEPHS2, SPS2	Involved in synthesis of all selenoproteins, including itself [100].	60	448
Iodothyronine deiodinase 1	DIO1, D1	Important for systemic active thyroid hormone levels [101].	126	249
Iodothyronine deiodinase 2	DIO2, D2	ER enzyme important for local active thyroid hormone levels [101].	133, 266	265
Iodothyronine deiodinase 3	DIO3, D3	Inactivates thyroid hormone [101].	144	278
Selenoprotein N	SELENON, SELN, SEPN1, SepN	Transmembrane protein localized to endoplasmic reticulum (ER). Mutations lead to multiminicore disease and other myopathies [102,103].	428	556
Selenoprotein P	SELENOP, SEPP1, SEP, SELP, SEPP	Secreted into plasma for selenium transport to tissues [77,104].	59, 300, 318, 330, 345, 352, 367, 369, 376, 378	381
Selenoprotein 15kDa	15kDa, SEP15	ER-resident thioredoxin-like oxidoreductase that complexes with uridine–guanosine–guanosine–thymodine (UGGT) and improves protein quality control by correcting misglycosylated/misfolded glycoproteins via the calnexin–calreticulinendoplasmic reticulum protein 57 (ERp57) axis and pH-dependent endoplasmic reticulum protein 44 (ERp44) system [105,106].	93	162
Selenoprotein M	SELENOM, SELM, SEPM	Thioredoxin-like ER-resident protein that may be involved in the regulation of body weight and energy metabolism [107].	48	145
Selenoprotein K	SELENOK, SELK	Transmembrane protein localized to the ER and involved in calcium flux in immune cells, as well as ER-associated degradation in cell lines [108,109].	92	94
Selenoprotein S	SELENOS, SELS, SEPS1, VIMP	Transmembrane protein found in the ER and involved in ER-associated degradation [110,111].	188	189
Selenoprotein O	SELENOO, SELO	Mitochondrial protein that contains a C-X-X-U motif (where C is cytosine, X is any nucleotide, and U is uridine), suggestive of redox function [112].	667	669
Selenoprotein W	SELENOW, SELW, SEPW1	Putative antioxidant role, which may be important in muscle growth [113].	13	87
Selenoprotein T	SELENOT, SELT	Oxidoreductase localized to the Golgi complex and ER and manifests a thioredoxin-like fold and is involved in redox regulation and cell anchorage. Complexes with UDP-glucose: glycoprotein glucosyltransferases to improve process quality control. Deficiency leads to early embryonic lethality [114].	36	182
Selenoprotein H	SELENOH, SELH, C11orf31	Nuclear localization, which is involved in redox sensing and transcription [115,116].	44	122
Selenoprotein V	SELENOV, SELV	Testes-specific expression [25].	273	346
Selenoprotein I	SELENOI, SELI, SPT1	Involved in phospholipid biosynthesis [117].	387	397

### 4.1. Antioxidant Properties of Selenium

The oxidation process in the body refers to a mechanism by which organic or inorganic peroxides cause damage to cells, thereby affecting their normal functions. Reactive Oxygen Species (ROS) are natural byproducts of oxygen metabolism and play a crucial role in cell signaling and maintaining homeostasis. During an organism’s metabolism, ROS are continuously generated in various organelles and through multiple metabolic pathways [118]. These species mainly include hydrogen peroxide (H_2_O_2_), hydroxyl radicals (OH^−^), superoxide anions (·O^2−^), and singlet oxygen (^1^O_2_). However, excessive production or metabolic imbalance of these highly reactive and unstable free radicals can lead to oxidative stress (OS). If not promptly eliminated, they can cause damage to the body, including lipid peroxidation, protein carbonylation, and DNA damage, potentially leading to various diseases.

**Figure 5:**
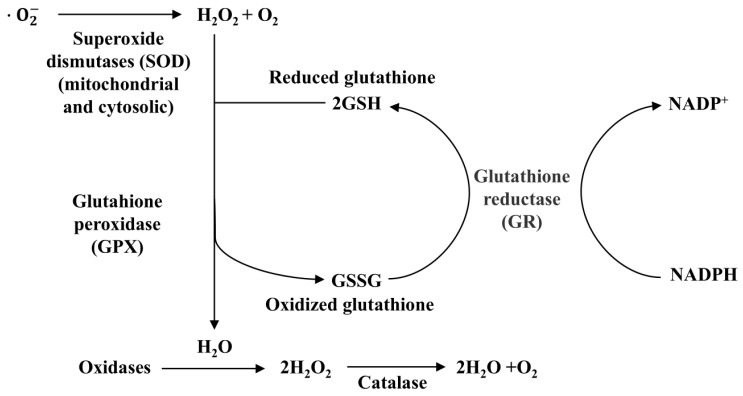
The role of catalase and glutathione peroxidase in the removal of hydrogen peroxide in the human body [55].

To maintain the balance of ROS, the antioxidant system within organisms comprises endogenous enzymes (such as GPX and TrxR) and exogenous antioxidants (such as Vitamin E). Selenium plays a role in synthesizing various selenoproteins, though not all possess antioxidant properties. Research indicates that nearly half of the selenoproteins have antioxidant functions, including GPXs, TrxRs, DIOs, Selenoprotein P, Selenoprotein M, Selenoprotein H, Selenoprotein O, and Selenoprotein V. Notably, as a vital component of GPX, selenium functions as an antioxidant by catalyzing the reduction of glutathione (GSH) into its oxidized form (GSSG), thereby converting toxic peroxides into non-toxic hydroxyl compounds. This process decomposes H_2_O_2_ into H_2_O, thus protecting cells and tissues from peroxide damage. It is particularly important for protecting the membranes of cells and organelles like mitochondria, microsomes, and lysosomes. The processes in which catalase and GPX enzymes eliminate hydrogen peroxide free radicals in human tissues are illustrated in Figure 5 [119].

**Figure 6:**
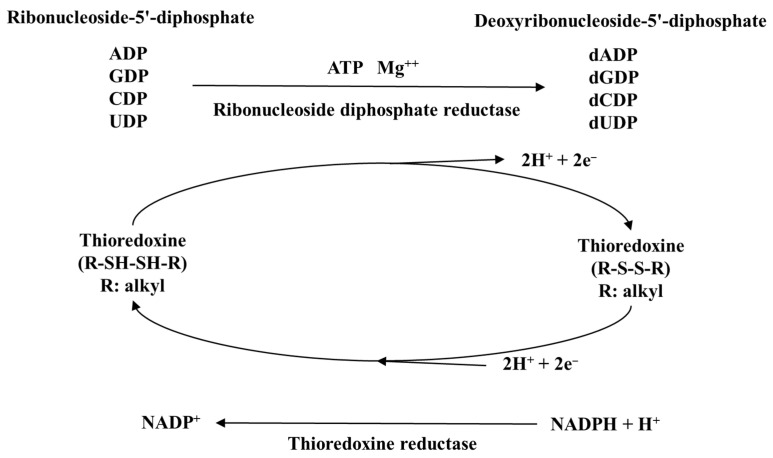
Role of thioredoxin in the reduction of ribonucleosides-5′-diphosphates to deoxyribonucleosides-5′-diphosphate.

TrxR includes three isoforms: TrxR1, which is widely distributed among various organelles and the cytoplasm; TrxR2, which is exclusively expressed in mitochondria; and TrxR3, which has testis-specific expression [120,121]. TrxR works together with thioredoxin (Trx) and reduced coenzyme II (NADPH) to form an antioxidant system [122,123]. It regulates redox reactions mediated by NADPH, reducing oxidized thioredoxin to its reduced form, thereby exerting its antioxidant function. Figure 6 demonstrates the specific role of thioredoxin in the reduction of ribonucleoside 5′-diphosphate to deoxyribonucleoside 5′-diphosphate. Trx is a widely distributed small dithiol protein that contains 104 amino acids, including one disulfide bridge, two SH groups, and a conserved active site (Trp-Cys-Gly-Pro-Cys) [119]. As a ubiquitously present redox protein, Trx can regulate various intracellular redox-related processes, reversibly reduce disulfide bonds, help reduce cellular oxidative stress by removing hydrogen peroxide, and enhance the cell’s antioxidant capacity [122,123]. Additionally, Trx plays a role in the reduction of ribonucleotides to deoxyribonucleotides, regulates enzymes and transcription factors through thiol redox control, and serves as a hydrogen donor for ribonucleotide reductase, which is crucial for the DNA synthesis necessary for repair mechanisms.

**Figure 7:**
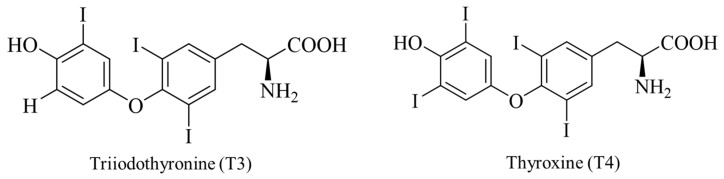
Formulae of thyroxine and triiodothyronine.

The antioxidant mechanism of DIOs is achieved by influencing the metabolism related to thyroid hormones [124]. Thyroid hormones primarily consist of triiodothyronine (T3) and thyroxine (T4), with their structural formulas shown in Figure 7. Among these, T4 does not possess hormonal activity, while T3 has very high hormonal activity and is the principal hormone exerting the effects of thyroid hormones. The synthesis of thyroid hormones is accompanied by the production of free radicals and various peroxides, especially H_2_O_2_. Selenium, as an essential component of the deiodinase enzymes that regulate thyroid hormones, aids in the conversion of T4 to T3 and participates in the regulation of thyroid hormone synthesis [125]. Additionally, it plays a role in the removal of oxidative substances, maintaining the balance between oxidation and antioxidation, thereby preventing oxidative damage to thyroid epithelial cells.

Selenoprotein P is the primary form of selenium present in plasma, accounting for more than half of the plasma selenium concentration [126]. It exerts antioxidant effects through three mechanisms: direct, indirect, and combined actions. In its direct action, selenoprotein P binds to heparin on the cell membrane surface and exhibits antioxidant effects against peroxides, such as peroxynitrite. The indirect action is achieved by promoting the expression and activity of other antioxidant enzymes, such as GPX and TrxR. Additionally, selenoprotein P can act in concert with these antioxidant enzymes to optimally maintain the stability of the body’s internal environment [127]. Other selenoproteins with antioxidant functions, such as selenoprotein M, primarily act as reductases within their respective pathways, reducing the production of reactive oxygen species or scavenging existing reactive oxygen species, thereby exerting direct or indirect antioxidant effects [127].

Additionally, selenium works synergistically with another antioxidant, vitamin E. Although vitamin E exerts its antioxidant effect by preventing the oxidation of unsaturated fatty acids into hydroperoxides, exogenous antioxidants form the second line of defense against free radical damage in the body. In recent years, domestic and international researchers have obtained various selenium compounds through in vivo enrichment and in vitro modification. Studies have shown that selenium compounds, such as selenium-enriched yeast, selenium polysaccharides, selenium nanoparticles, and exogenous selenium proteins, all exhibit significant antioxidant activity [58,59].

### 4.2. Regulation of the Immune System by Selenium

The human immune system comprises two parts: nonspecific immunity and specific immunity. Nonspecific immunity is an innate physiological defense mechanism that relies on phagocytic cells (such as macrophages and neutrophils) to engulf and dissolve invading pathogens, thereby maintaining health. Macrophages, while performing their phagocytic function, can be harmed by peroxides. However, the enzyme glutathione peroxidase within the human body can mitigate this damage, thereby protecting the macrophages. Supplementing with selenium can help enhance the phagocytic and bactericidal capabilities of these cells, thereby improving nonspecific immune function [128,129].

Specific immunity is acquired adaptive immunity that the body develops after encountering and combating foreign microorganisms. It is primarily mediated through B cells (humoral immunity) and T cells (cell-mediated immunity). In humoral immunity, selenium boosts the differentiation, proliferation, and antibody production of lymphocytes, thus enhancing the formation of immunoglobulins such as IgM and IgG. Selenium deficiency can inhibit the production of immunoglobulins and antibodies. Selenium also promotes the proliferation and cytotoxic function of T cells, enhances antibody production by B cells, bolsters antiviral capability, and suppresses viral activity. In specific immune responses, humoral immunity and cell-mediated immunity both have their unique roles but can also cooperate to exert overall immune effects.

Selenium is most abundantly found in tissues such as lymph nodes, liver, and spleen, which are rich in immune cells. Experts have discovered that selenium is widely present in all immune cells and plays a role in protecting the thymus, maintaining lymphocyte activity, and promoting antibody formation. Supplementing with selenium helps to enhance the body’s immune system and defend against various diseases. In summary, selenium deficiency can impact all aspects of the immune system, while selenium supplementation can improve cellular immunity, humoral immunity, and nonspecific immune functions. One possible mechanism for this action is the enhancement of selenium-containing GPX activity, which reduces the accumulation of lipid peroxides in immune cells, thereby enhancing immune cell function [130].

### 4.3. Selenium Promotes the Synthesis of Thyroid Hormones

**Figure 8:**
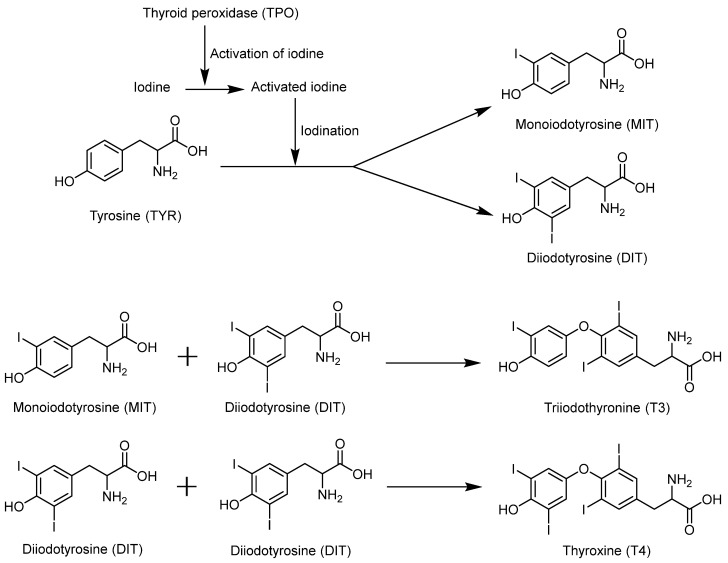
The process of thyroid hormone synthesis.

The thyroid gland is the largest endocrine gland in the human body. It secretes thyroid hormones (THs) that can affect almost all cells and play important roles in regulating growth, development, and metabolism. The primary thyroid hormones include triiodothyronine (T3) and thyroxine (T4), and the thyroid gland is the only source of these hormones in the body. Figure 8 shows the synthesis process of thyroid hormones. Thyroid follicular epithelial cells use thyroid peroxidase (TPO) to activate the iodine that has been ingested. Activated iodine then iodizes tyrosine residues in thyroglobulin (TG) to form monoiodotyrosine (MIT) or diiodotyrosine (DIT). MIT and DIT couple to form T3, while two DIT molecules couple to form T4.

**Figure 9:**
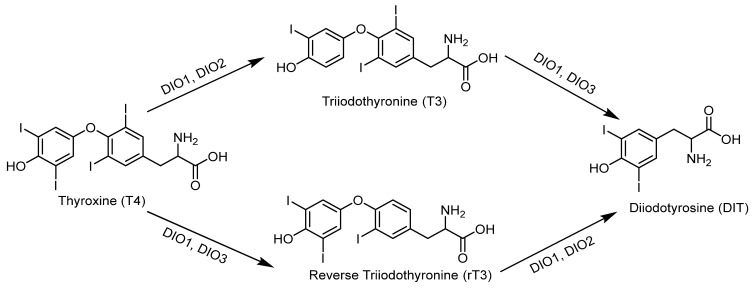
The role of deiodinases in the conversion between different thyroid hormones.

The thyroid gland expresses various selenoproteins, including DIO1, DIO2, GPX1, GPX3, GPX4, TrxR1, TrxR2, TrxR3, as well as selenoprotein F, selenoprotein P, and selenoprotein M [131]. These selenoproteins play crucial roles in the production of thyroid hormones and in maintaining their stability in the body. GPX and TrxR regulate the efficiency of thyroid hormone biosynthesis by participating in cellular oxidation reactions. DIO includes three types of deiodinases (DIO1, DIO2, DIO3). DIO1 is mainly present in the liver, kidneys, and thyroid; DIO2 is found in the pituitary gland, thyroid, and skeletal muscle; while DIO3 is mainly distributed in the cerebral cortex, placenta, and skin tissues and is absent in the thyroid [132,133]. The three types of deiodinases collectively regulate the interconversion between different active forms of thyroid hormones in the body, thereby maintaining balance [134]. The role of deiodinases in this interconversion process is shown in Figure 9.

DIO1, DIO2, and DIO3 have different roles in the conversion of thyroid hormones. Specifically, DIO1 has a bidirectional effect on thyroid hormones: it can promote the conversion of low bioactive T4 to highly bioactive T3 and facilitate the conversion of highly bioactive T3 to low bioactive DIT [110]. DIO2 mainly catalyzes the outer ring deiodination to convert T4 into T3. DIO3 primarily inhibits T3 through inner ring deiodination, converting T4 into inactive rT3 or DIT [110,135]. The biological activity of T3 is approximately five times that of T4, but only about 20% of T3 is directly secreted by the thyroid; the majority of T3 is derived from the deiodination of T4 in peripheral tissues [136], making deiodinases crucial in the regulation of TH. When the body is deficient in selenium, the thyroid prioritizes the retention, redistribution, and increased expression of specific selenoproteins. Upon selenium supplementation, there is a preferential accumulation of selenium in the thyroid [137]. In conclusion, selenium plays an essential role in maintaining normal body function and thyroid functionality.

### 4.4. Other Biological Functions of Selenium

In addition to the aforementioned biological functions, selenium has been proven to prevent tumor occurrence, reduce cardiovascular disease mortality, prevent and treat diabetes, prevent neurodegenerative diseases, enhance male fertility, and control inflammatory responses [138,139]. In the 1970s, scientists discovered the anti-tumor effects of selenium. Epidemiological studies have also shown that blood selenium levels in populations determined by geological environment and diet are negatively correlated with the incidence and mortality of tumors [140]. Multiple studies have confirmed that the incidence of cancer is closely related to selenium deficiency. Selenium can prevent tumor occurrence, inhibit tumor growth, promote tumor cell differentiation, inhibit cell division, and reverse malignant phenotypes. Although the mechanisms of selenium’s anti-tumor effects are not yet fully understood, it is believed that selenium’s anti-tumor effects are partly achieved by regulating and enhancing the body’s immune function, as the immune system is closely related to the occurrence and development of tumors. Some viewpoints suggest that the anti-cancer effects of selenium result from a combination of multiple mechanisms. As regulatory agents, organic selenium compounds can influence the expression of oncogenes and induce programmed cell death in cancer cells while also affecting cellular immune functions. Organic selenium compounds can regulate various developmental patterns of tumor cells either directly or indirectly. Angiogenesis is a critical step in the development and metastasis of solid tumors. Increasing evidence indicates that selenium compounds have a significant inhibitory effect on tumor angiogenesis. Vascular endothelial growth factor (VEGF) is a key protein that stimulates angiogenesis. Numerous studies have demonstrated that selenium compounds can regulate the expression of VEGF and related angiogenic factors, thereby inhibiting tumor angiogenesis [141,142,143].

The pathological basis of various cardiovascular diseases, such as coronary heart disease and hypertension, is atherosclerosis [144]. Epidemiological studies and clinical observations have found that selenium has anti-atherosclerotic effects. Selenium supplementation can reduce the formation of atherosclerosis both quantitatively and qualitatively. Meanwhile, selenium-containing antioxidant enzymes in myocardial tissue are involved in the removal of H_2_O_2_ in myocardial cells, thereby protecting the proper function of cellular organelles such as the myocardial cell membrane and mitochondria. Additionally, research has shown that selenium content in the diet is negatively correlated with hypertension [19].

A substantial body of research indicates a negative correlation between selenium and blood glucose levels [145,146]. However, the causal relationship between hyperglycemia and low blood selenium levels remains controversial. One perspective suggests that oxidative stress induced by elevated blood glucose consumes GPX, leading to a reduction in serum selenium concentration. Conversely, another viewpoint posits that a decrease in serum selenium leads to reduced levels of GPX in the body, weakening antioxidant capacity, enhancing lipid peroxidation, and ultimately damaging pancreatic β-cells, thereby triggering diabetes. Selenium, as an active component of the GPX enzyme, can protect the pancreas from oxidative damage induced by streptozotocin to a certain extent, thereby restoring its function and improving symptoms in patients with Type II diabetes. This provides a basis for the prevention and treatment of diabetes.

Researchers have found that selenium and selenium compounds play significant roles in neurotransmission pathways involving γ-aminobutyric acid (GABA) neurons [147], dopaminergic neurons, cholinergic neurons, and glutaminergic neurons. These compounds can effectively prevent the onset of neurodegenerative diseases such as Alzheimer’s disease, Parkinson’s disease, and amyotrophic lateral sclerosis (ALS) [148,149,150]. Additionally, selenium is closely related to reproductive health, especially in males, affecting testicular tissue, the quantity of spermatogonia, sperm formation, sperm morphology, and libido. Severe selenium deficiency can lead to male infertility [151,152,153]. Furthermore, selenium supplementation can prevent bone marrow lesions and promote repair, offering preventive and therapeutic benefits for conditions like Keshan disease, Kashin–Beck disease, and arthritis [154,155]. Thus, selenium and its compounds can act on multiple organs and tissues in the body, improving their functions and demonstrating extensive biological activity.

## 5. Conclusions

Selenium, an essential trace element for the human body, plays multiple critical biological roles. Its antioxidant properties protect cells from oxidative stress damage, thereby reducing the risk of chronic diseases such as cardiovascular diseases and certain cancers. Selenium is also integral to the normal functioning of the thyroid and the immune system, making adequate intake crucial for maintaining these physiological processes. However, excessive intake can lead to adverse health effects, including selenium toxicity. Therefore, maintaining an appropriate level of selenium intake is vital, which underscores the importance of a well-balanced diet and the cautious use of supplements. Future research should focus on exploring the specific requirements and mechanisms of selenium in different populations to better inform dietary recommendations and public health policies. Additionally, the biological mechanisms by which selenium contributes to human health warrant further study. In conclusion, as a vital factor for human well-being, balanced selenium intake deserves significant attention.

## Figures and Tables

**Table 2:** The selenium content in some foods [61].

Food Categories	Selenium Content (mg/kg)
Grains and grain products	0.01–0.55
Meat, fish, eggs	0.01–0.36
Milk and milk products	<0.001–0.17
Vegetables and fruits	<0.001–0.022
Bovine kidney	0.78–1.45
Brazil nuts	0.83–53
Cabbage	<0.001–0.46
Asparagus	0.01–1.40

**Table 3:** Daily selenium intake, selenium levels in human serum, urine and breast milk, and soil selenium concentrations in several countries [62,63,64,65,66].

Country	Intake (μg/day)	Serum (μg/L)	Breast Milk (μg/L)	Urine (μg/L)	Soil (mg/kg)
Belgium	28–61	73–110	9.7–153	13–30	0.11
Brazil	60	/	14.1	/	/
China (Enshi Province)	3200–6690	1300–7500	94.8–120.5	2680	10–40
China (Keshan region)	3–11	23.9	3.0	7	0.17
Finland	125	77–134	6–14.3	/	0.15–0.72
France	47	84.7	/	12.3	0.18
Germany	47	63–106	9.9–59	16–23	6.6
Italy	49	76–94	13.3	7.4	/
Japan	133	/	11.2–40.3	36–288	0.7–1.0
Spain	60	74–84	11.4–21.7	/	0.07–0.39
Sweden	38	105	13.1	36	0.39
Switzerland	70	96–113	/	/	/
Turkey	30	58–113	11.2–48.6	/	0.03
Netherlands	67	93.6	/	/	/
UK	41	60–81	8.3	5	0.18–29.70
USA	98	95–320	7–105	19.2–118	0.11–18.36

“/” no date available.

**Table 4:** The Chinese Nutrition Society’s recommended estimated average requirement (EAR), recommended nutrient intake (RNI), and tolerable upper intake level (UL) of selenium for different populations.

Age	EAR (μg/d)	RNI (μg/d)	UL (μg/d)
0 to under 6 months	/	15 (AI)	55
6 to under 12 months	/	20 (AI)	80
1 to under 4 years	20	25	100
4 to under 7 years	25	30	150
7 to under 11 years	35	40	200
11 to under 14 years	45	55	300
14 to under 18 years	50	60	350
18 to under 50 years	50	60	400
50 years and older	50	60	400
Pregnant women	54	65	400
Lactating women	65	78	400

“/” no date available; “AI” is adequate intake.

**Table 5:** The recommended daily selenium intake (μg/d) for residents by other countries and the WHO.

Age	USA [67]	EU [68]	Canada [69]	UK [70]	New Zealand [71]	Germany [72]	Austria [72]	Switzerland [72]	Australian [73]	WHO [74]
0 to under 4 months	/	/	/	/	/	10	10	10	10	6
4 to under 12 months	/	/	/	/	/	15	15	15	15	10
1 to under 4 years	20	/	/	/	/	15	15	15	25	17
4 to under 7 years	30	/	/	/	/	20	20	20	30	22
7 to under 10 years	30	/	/	/	/	30	30	30	50	21
10 to under 13 years (male)	40	/	/	/	/	45	45	45	50	32
10 to under 13 years (female)	555	/	/	/	/	45	45	45	50	26
13 to under 15 years (male)	55	/	/	/	/	60	60	60	85	34
13 to under 15 years (female)	55	/	/	/	/	60	60	60	85	26
15 to under 19 years (male)	55	/	/	/	/	70	70	70	85	34
15 to under 19 years (female)	55	/	/	/	/	60	60	60	85	26
19 to under 65 years (male)	55	55	55	75	60	70	70	70	60	34
19 to under 65 years (female)	55	55	55	60	55	60	60	60	55	26
65 years and older (male)	55	/	/	/	/	70	70	70	/	33
65 years and older (female)	55	/	/	/	/	60	60	60	/	30
Pregnant women	49	/	/	/	/	60	60	60	80	29
Lactating women	59	/	/	/	/	75	75	75	85	39

“/” no date available.

## Data Availability

Not applicable.
